# Vital Signs: Carbapenem-Resistant Enterobacteriaceae

**Published:** 2013-03-08

**Authors:** Jesse T. Jacob, Eili Klein, Ramanan Laxminarayan, Zintars Beldavs, Ruth Lynfield, Alexander J. Kallen, Philip Ricks, Jonathan Edwards, Arjun Srinivasan, Scott Fridkin, J. Kamile Rasheed, David Lonsway, Sandie Bulens, Rosa Herrera, L. Clifford McDonald, Jean Patel, Brandi Limbago, Michael Bell, Denise Cardo

**Affiliations:** Emory Univ School of Medicine, Atlanta, Georgia; Center for Advanced Modeling, Dept of Emergency Medicine, Johns Hopkins Univ, Baltimore, Maryland; Center for Disease Dynamics, Economics, and Policy, District of Columbia; Oregon Health Authority; Minnesota Dept of Health; Div of Healthcare Quality Promotion, National Center for Emerging and Zoonotic Diseases, CDC

## Abstract

**Background:**

Enterobacteriaceae are a family of bacteria that commonly cause infections in health-care settings as well as in the community. Among Enterobacteriaceae, resistance to broad-spectrum carbapenem antimicrobials has been uncommon. Over the past decade, however, carbapenem-resistant Enterobacteriaceae (CRE) have been recognized in health-care settings as a cause of difficult-to-treat infections associated with high mortality.

**Methods:**

The percentage of acute-care hospitals reporting at least one CRE from health-care–associated infections (HAIs) in 2012 was estimated using data submitted to the National Healthcare Safety Network (NHSN) in 2012. The proportion of Enterobacteriaceae infections that were CRE was calculated using two surveillance systems: 1) the National Nosocomial Infection Surveillance system (NNIS) and NHSN (for 2001 and 2011, respectively) and 2) the Surveillance Network–USA (TSN) (for 2001 and 2010). Characteristics of CRE culture-positive episodes were determined using data collected as part of a population-based CRE surveillance project conducted by the Emerging Infections Program (EIP) in three states.

**Results:**

In 2012, 4.6% of acute-care hospitals reported at least one CRE HAI (short-stay hospitals, 3.9%; long-term acute-care hospitals, 17.8%). The proportion of Enterobacteriaceae that were CRE increased from 1.2% in 2001 to 4.2% in 2011 in NNIS/NHSN and from 0% in 2001 to 1.4% in 2010 in TSN; most of the increase was observed in *Klebsiella* species (from 1.6% to 10.4% in NNIS/NHSN). In the EIP surveillance, 92% of CRE episodes occurred in patients with substantial health-care exposures.

**Conclusions:**

Carbapenem resistance among common Enterobacteriaceae has increased over the past decade; most CRE are associated with health-care exposures.

**Implications for Public Health:**

Interventions exist that could slow the dissemination of CRE. Health departments are well positioned to play a leading role in prevention efforts by assisting with surveillance, situational awareness, and coordinating prevention efforts.

## Introduction

The Enterobacteriaceae are a large family of gram-negative bacilli that are normal inhabitants of the gastrointestinal tract of humans and other animals (1). These organisms are a common cause of community-acquired and health-care–acquired infections. Although this family includes more than 70 genera, the health-care–associated Enterobacteriaceae most commonly reported to CDC’s National Healthcare Safety Network (NHSN) surveillance system are *Escherichia coli*, *Klebsiella* species, and *Enterobacter* species (2). The past several decades have seen the spread of Enterobacteriaceae with resistance to broad-spectrum antimicrobials; however, clinicians in the United States have relied on the carbapenem antimicrobial class (imipenem, meropenem, doripenem, and ertapenem) to treat infections caused by these resistant organisms. Carbapenem-resistant Enterobacteriaceae (CRE) were relatively uncommon in the United States before 2000 (3). Unlike resistance in methicillin-resistant *Staphylococcus aureus* (MRSA), which is one bacterial species and is mediated by a single mechanism, carbapenem resistance is complex; it can occur in different Enterobacteriaceae and be mediated by several mechanisms, including production of enzymes that inactivate carbapenems (carbapenemases). *Klebsiella pneumoniae* carbapenemase (KPC), an enzyme encoded by a highly transmissible gene, was first identified from a *Klebsiella* isolate in 2001 (4) and has now spread widely throughout the United States and around the world. In addition to KPC, a number of additional carbapenemases that have emerged among Enterobacteriaceae outside the United States (e.g., New Delhi metallo-beta-lactamase [NDM]) have been identified in this country. CRE can spread in health-care settings and cause infections with mortality rates of 40% to 50% (5–7). In this report, recent changes in the epidemiology and incidence of CRE in the United States are described.

## Methods

The objectives of this evaluation were to 1) describe the extent of CRE spread among acute-care hospitals, 2) estimate the proportion of clinical isolates of Enterobacteriaceae that are resistant to carbapenems in the United States, and 3) determine characteristics of CRE culture-positive episodes. Because no single surveillance system includes all the data required for these analyses, data from three systems are included in this report. CRE definitions used for objectives 1 and 2 were slightly different than that used for objective 3 because of the use of these different systems.

The first objective was accomplished using NHSN data for the first 6 months of 2012. All facilities performing surveillance for central-line–associated bloodstream infections (CLABSIs) or catheter-associated urinary tract infections (CAUTIs) were reviewed for reports of CRE isolates, defined as *E. coli*, *Klebsiella pneumoniae*, *Klebsiella oxytoca*, *Enterobacter cloacae*, or *Enterobacter aerogenes* that were nonsusceptible to imipenem, meropenem, or doripenem.

For the second objective, data from NHSN and its predecessor, the National Nosocomial Infection Surveillance system (NNIS), were used. Intensive-care unit (ICU) CLABSIs, ICU CAUTIs, and surgical site infections after colon surgery or coronary artery bypass grafting reported to NNIS in 2001 or NHSN in 2011 for which an isolate of one of the Enterobacteriaceae listed above was reported were included. To evaluate infections across another set of isolates collected hospital-wide, a similar analysis was performed by the Center for Disease Dynamics, Economics, and Policy, using data from the Surveillance Network-USA (TSN) (managed by Eurofins Medinet; Chantilly, Virginia). TSN is an electronic repository of susceptibility test results collected from approximately 300 laboratories that are selected to be demographically representative of the United States at the level of the nine U.S. Census regions (8). Similar definitions were used for the TSN analysis; however, *K. oxytoca* was not included, and surveillance periods included 2001 and the first 6 months of 2010.

The third objective was accomplished using data collected during the internally funded pilot of a population-based CRE surveillance project conducted through CDC’s Emerging Infections Program (EIP) at three sites (Atlanta, Georgia; Minneapolis-St. Paul, Minnesota; and Portland, Oregon metropolitan areas). Laboratories were asked for reports of CRE, defined in this report as Enterobacteriaceae from sterile-site and urine cultures that were nonsusceptible to imipenem, meropenem, or doripenem and resistant to all third-generation cephalosporins tested (e.g., ceftriaxone, cefotaxime, and ceftazidime). Resistance to third-generation cephalosporins was included in this surveillance system to increase the specificity for carbapenemase-producing Enterobacteriaceae. Medical records for CRE patients were reviewed. CRE-positive clinical cultures were classified as hospital-onset if the culture was taken from a hospital inpatient after the third day of admission. A health-care exposure was defined as a recent (i.e., within the past year) hospitalization, long-term–care admission, surgery, dialysis, or the presence of an indwelling device in the 2 days before the positive culture.

## Results

During the first 6 months of 2012, among the 3,918 U.S. acute-care hospitals performing surveillance for either CAUTI or CLABSI in any part of their hospital, 181 (4.6%) reported one or more infections with CRE (145 [3.9%] in short-stay hospitals; 36 [17.8%] in long-term acute-care hospitals [LTACHs]). The percentage of facilities with CRE was stratified by selected characteristics; of note, the percentage of hospitals reporting CRE was highest in the Northeast and among larger and teaching hospitals (Table 1).

The percentage of Enterobacteriaceae that were CRE reported to NNIS in 2001 was 1.2%; in NHSN in 2011, it was 4.2%. The proportion CRE varied by organism and increased most for *Klebsiella* species, from 1.6% to 10.4% (Table 2). Data from TSN demonstrated an increase from 0% to 1.4%, with the largest increase among *K. pneumoniae* (0% to 5.3%).

During the 5-month EIP project pilot, 72 CRE were identified from 64 patients (56 patients had one positive culture; eight had two). Most came from the Atlanta metropolitan area (59) followed by Minneapolis-St. Paul (10), and Portland (three). Most CRE were *Klebsiella* species (49) followed by *Enterobacter* species (14) and *E. coli* (nine). The most common source was urine (89%), followed by blood (10%). CRE culture-positive episodes were stratified by selected characteristics (Table 3). Most isolates were from cultures collected outside of acute-care hospitals (47 of 71); however, most of these community-onset isolates were from patients with health-care exposures (41 of 47), particularly recent hospitalization (72%).

## Conclusions and Comment

Although CRE remain relatively uncommon in most acute-care hospitals in the United States, they have become an increasingly recognized cause of infection during the past decade, especially among *Klebsiella*, likely because of the emergence of carbapenemase-producing strains. In 2012, the number of facilities reporting CRE as a cause of infection was small, and spread of these organisms appears to be uneven both regionally and among facilities within regions. Fewer than 5% of short-stay acute-care hospitals reported CRE from health-care–associated infections in the first half of 2012; CRE more often were reported from LTACHs. Data from population-based surveillance suggest most CRE clinical isolates came from cultures collected outside of hospitals from patients with substantial health-care exposures. These findings suggest that although CRE are increasing in prevalence, their distribution is limited.

CRE are important for several reasons. First, invasive infections (e.g., bloodstream infections) with CRE are associated with mortality rates exceeding 40% (5); this is significantly higher than mortality rates observed for carbapenem-susceptible Enterobacteriaceae. Of note, because the majority of positive cultures were from urine, overall in-hospital mortality rates associated with positive cultures were lower in the EIP CRE surveillance (4%). Second, carbapenem-resistant strains frequently possess additional resistance mechanisms that render them resistant to most available antimicrobials; pan-resistant CRE have been reported (9). Further, novel antimicrobials for multidrug-resistant gram-negative bacilli are in early stages of development and not likely to be available soon (10). Third, CRE can spread rapidly in health-care settings (11,12). Fourth, Enterobacteriaceae are a common cause of community infections, and CRE have the potential to move from their current niche among health-care–exposed patients into the community (13). Multidrug-resistance is a problem in other gram-negative bacilli such as *Pseudomonas* and *Acinetobacter* species. However, these organisms are a less common cause of health-care infections and have less potential to spread resistance to other bacteria and into the community (2).

Current CRE prevention strategies are based on the identification of patients colonized or infected with CRE followed by implementation of contact precautions. Colonization commonly is detected through rectal surveillance cultures of patients at risk for CRE (e.g., patients exposed to known cases of CRE). Active case detection and immediate implementation of interventions, often including cohorting staff and CRE patients (i.e., segregating CRE-colonized or CRE-infected patients and the health-care personnel who care for them from those without CRE and the health-care personnel who care for them), has been used successfully to control CRE in acute-care and long-term–care settings (6,7,14). Efforts to ensure appropriate antibiotic use in hospitals and nursing homes also are critical to slowing CRE emergence.* Patients who are colonized or infected with CRE often are cared for in multiple types of health-care institutions during their illnesses. Therefore, having a broader, multi-institutional or regional approach to prevention is necessary for control, particularly in regions where CRE are just beginning to be recognized. Regional efforts to control multidrug-resistant organisms (MDROs) have been employed successfully, including a coordinated effort to control vancomycin-resistant *Enterococcus* in the Siouxland region of Iowa, Nebraska, and South Dakota (15) and a national response to MRSA in the Netherlands (16). For CRE, Israel has effectively employed a nationwide coordinated control effort since KPC-producing strains emerged there in 2006 (6).

State and local health departments are well positioned to lead CRE control efforts because of their expertise in surveillance and prevention and their ability to interact among all the health-care facilities in their jurisdiction. To date, many health departments have conducted surveillance efforts in an attempt to identify the CRE incidence in their region (17).† In addition, six states have made CRE reportable, and three additional states are actively pursuing this option. Requiring CRE reporting can allow for a better understanding of the changing CRE burden and can help facilitate intervention. Beyond surveillance, several states have developed and implemented plans to assist health-care facilities with control efforts when CRE are identified. As new MDROs emerge over time, this regional approach to MDRO prevention has implications beyond CRE as well.

The findings in this report are subject to at least three limitations. First, antimicrobial susceptibility data reported to NNIS and NHSN were generated at individual institutions rather than a central laboratory, and testing methodologies vary between facilities. Second, susceptibility interpretation is based on the recommended breakpoints used when tested. Although carbapenem breakpoints for Enterobacteriaceae were lowered in 2010 (18) and might have influenced the increase in the percentage of isolates that were carbapenem-resistant, most laboratories would not have incorporated those changes by 2011. Finally, in some instances, complete susceptibility test results, particularly for carbapenems, were not reported to NNIS or NHSN, leading to a subset of isolates that were not included in these analyses. Not reporting results for carbapenems would be more likely when organisms were susceptible to less broad-spectrum antimicrobials; therefore, many of the organisms for which carbapenem susceptibility information was not available might have been susceptible. As a result, the percentage resistant reported from NNIS and NHSN likely represents an overestimate of the actual percentage resistant; however, the proportion of NHSN facilities reporting at least one CRE should not be affected.

The high proportion of LTACHs with CRE in 2012 highlights the need to expand prevention outside of short-stay acute-care hospitals into settings that, historically, have had less developed infection prevention programs. Additional research is needed to clarify unanswered questions, including assessing which CRE prevention strategies are most effective and investigating new prevention approaches such as decolonization. Fortunately, many regions are in a position to prevent the further emergence of these organisms if they act aggressively. To do so will require expanded and coordinated action from clinicians, facility administrators, and public health officials.

**Key Points**

- Enterobacteriaceae are gram-negative bacteria (e.g., *Klebsiella, Proteus, Serratia, Enterobacter, and Escherichia coli*) that can cause invasive disease but generally have been susceptible to a variety of antibiotics. Carbapenem-resistant Enterobacteriaceae (CRE) are Enterobacteriaceae that have become highly resistant to most or all antibiotics through several mechanisms. Carbapenem resistance, while relatively uncommon among Enterobacteriaceae (observed in about 4% of Enterobacteriaceae in this study), has increased from about 1% during the past decade. CRE bloodstream infections are associated with mortality rates approaching 50%.
- CRE has now spread throughout the United States but in most areas they remain relatively uncommon; about 4% of acute-care hospitals and 18% of long-term acute-care hospitals reported at least one CRE to the National Healthcare Safety Network in the first 6 months of 2012. Nearly all patients with CRE were currently or recently treated in a health-care setting. However, CRE could spread into the community among otherwise healthy persons.
- Preventing spread is important before CRE gains a foothold in more hospitals or in the community. This requires active case detection and contact precautions for colonized or infected patients as well as cohorting of patients and staff; appropriate antibiotic use in all settings; and communication about infections when patients transfer. Regional and state-based approaches have been shown to be effective in reducing incidence.
- Additional information is available at http://www.cdc.gov/vitalsigns.

## Figures and Tables

**TABLE 1:** Number and percentage of facilities reporting carbapenem-resistant* Enterobacteriaceae† from a catheter-associated urinary tract infection (CAUTI) or a central-line–associated bloodstream infection (CLABSI), by selected characteristics — United States, National Healthcare Safety Network, January–June 2012

Characteristic	No. of facilities with carbapenem-resistant Enterobacteriaceae from CAUTI or CLABSI	Total no. of facilities performing CAUTI or CLABSI surveillance (N = 3,918)	(%)§¶
**Facility type**			
All acute-care hospitals	181	3,918	(4.6)
Short-stay acute-care hospital	145	3,716	(3.9)
Long-term acute-care hospital	36	202	(17.8)
**Hospital size (no. of beds)**			
<100	48	1,609	(3.0)
100–299	46	1,480	(3.1)
300–499	41	541	(7.6)
≥500	45	258	(17.4)
**Medical school affiliation**			
Yes	102	1,079	(9.5)
No	53	2,839	(1.9)
**U.S. Census region** **			
Northeast	63	658	(9.6)
Midwest	30	927	(3.2)
South	50	1,503	(3.3)
West	29	804	(3.6)
Other††	9	26	(34.6)

* Intermediate or resistant to imipenem, meropenem, or doripenem.

† *Klebsiella pneumoniae*, *Klebsiella oxytoca*, *Escherichia coli*, *Enterobacter aerogenes*, or *Enterobacter cloacae.*

§ Total percentage of facilities performing any surveillance for any CAUTI and CLABSI during the first 6 months of 2012.

¶ For each category, p<0.01 by chi-square test.

** *Northeast*: Connecticut, Maine, Massachusetts, New Hampshire, New Jersey, New York, Pennsylvania, Rhode Island, and Vermont; *Midwest*: Illinois, Indiana, Iowa, Kansas, Michigan, Minnesota, Missouri, Nebraska, North Dakota, Ohio, South Dakota, and Wisconsin; *South*: Alabama, Arkansas, Delaware, District of Columbia, Florida, Georgia, Kentucky, Louisiana, Maryland, Mississippi, North Carolina, Oklahoma, South Carolina, Tennessee, Texas, Virginia, and West Virginia; *West*: Alaska, Arizona, California, Colorado, Hawaii, Idaho, Montana, Nevada, New Mexico, Oregon, Utah, Washington, and Wyoming.

†† Armed Forces, Puerto Rico, and U.S. Virgin Islands.

**TABLE 2:** Number of Enterobacteriaceae isolates, percentage reported to be tested against carbapenems, and percentage reported as carbapenem-resistant,* by data source, year, and type of organism — United States, National Nosocomial Infections Surveillance system (NNIS), National Healthcare Safety Network (NHSN), and the Surveillance Network–USA (TSN)†

Type of organism	NNIS (2001)			NHSN (2011)		
	
	No. of isolates	Reported as tested against ≥1 carbapenem No. (%)	Reported as carbapenem-resistant* No. (%)	No. of isolates	Reported as tested against ≥1 carbapenem No. (%)	Reported as carbapenem-resistant* No. (%)
*Klebsiella pneumoniae* and *oxytoca*	654	253 (38.7)	4 (1.6)	1,902	1,312 (69.0)	136 (10.4)
*Escherichia coli*	1,424	421 (29.6)	4 (1.0)	3,626	2,348 (64.8)	24 (1.0)
*Enterobacter aerogenes* and *cloacae*	553	288 (52.1)	4 (1.4)	1,045	728 (69.7)	26 (3.6)
**Total**	**2,631**	**962 (36.6)**	**12 (1.2)**	**6,573**	**4,388 (66.8)**	**186 (4.2)**

**Type of organism**	**TSN (2001)**			**TSN (2010)** §		
	
	**No. of isolates**	**Reported as tested against ≥1 carbapenem No. (%)**	Reported as carbapenem-resistant* **No. (%)**	**No. of isolates**	**Reported as tested against ≥1 carbapenem No. (%)**	Reported as carbapenem-resistant* **No. (%)**

*Klebsiella pneumoniae*	19,522	19,522 (100.0)	0 —	11,155	11,155 (100.0)	593 (5.3)
*Esherichia coli*	47,603	47,603 (100.0)	0 —	31,890	31,890 (100.0)	32 (0.1)
*Enterobacter aerogenes* and *cloacae*	14,764	14,764 (100.0)	3 (0)	5,768	5,768 (100.0)	69 (1.2)
**Total**	**81,889**	**81,889 (100.0)**	**3 (0)**	**48,813**	**48,813 (100.0)**	**694 (1.4)**

* Intermediate or resistant to imipenem, meropenem, or doripenem.

† NNIS and NHSN include Enterobacteriaceae reported from hospital infections (i.e., intensive-care unit central-line–associated bloodstream infections, intensive-care unit catheter-associated urinary tract infections, and surgical site infections after colon surgery or coronary artery bypass grafting). TSN includes Enterobaceriaceae isolates from clinical cultures from acute-care hospitals submitted to participating laboratories.

§ Includes isolates reported during January–June 2010.

**TABLE 3:** Number and percentage of episodes of positive cultures for carbapenem-resistant* *Enterobacteriaceae*† (N = 72) from three communities,§ by selected characteristics — United States, Emerging Infections Program, August–December 2011

Characteristic	No.	(%)
**Patient characteristics**		
Female sex	36	(50)
White race	32	(45)
Median age (range) (yrs)	60	(8–91)
<18	2	(3)
≥65	30	(42)
**Type of health-care exposure** ¶		
Hospitalization	34	(72)
Presence of urinary catheter within the past 2 days	22	(47)
Long-term care facility	17	(36)
Surgery	12	(26)
Presence of other indwelling device within the past 2 days	11	(23)
Presence of central line within the past 2 days	9	(19)
None	6	(4)
Dialysis	3	(13)
**Outcome**		
Hospitalized	59	(82)
Intensive-care unit within 7 days of positive culture	16	(22)
Died	3	(4)

* Nonsusceptible to imipenem, meropenem, or doripenem and resistant to all third-generation cephalosporins tested (e.g., ceftriaxone, cefotaxime, ceftazidime).

† *Klebsiella pneumoniae*, *Klebsiella oxytoca*, *Escherichia coli*, *Enterobacter aerogenes*, or *Enterobacter cloacae.*

§ Atlanta, Georgia; Minneapolis-St. Paul, Minnesota; and Portland, Oregon.

¶ Within the past year, unless noted otherwise, among community-onset cultures (n=47).

## Footnotes

* Detailed prevention recommendations for acute-care and long-term–care facilities are available at http://www.cdc.gov/hai/organisms/cre/cre-toolkit.

† An example of a survey that has been adapted by health departments to evaluate CRE incidence and CRE prevention activities in a region is available at http://www.cdc.gov/hai/organisms/cre/cre-toolkit/index.html.
